# Classification of impacted mandibular third molars on cone-beam CT images

**DOI:** 10.4317/jced.51984

**Published:** 2015-04-01

**Authors:** Michele Maglione, Fulvia Costantinides, Gabriele Bazzocchi

**Affiliations:** 1 MD, DDS, MSc, Associate Professor, Unit of Oral Surgery, School of Dental Sciences, Department of Medical Sciences, Surgery and Health, Trieste, Italy; 2 DDS, MSC, Adjunct Professor, Unit of Oral Surgery, School of Dental Sciences, Department of Medical Sciences, Surgery and Health, Trieste, Italy; 3 MD, MSc, PhD, Clinician, Unit of Radiology, “Maggiore” University Hospital, Trieste, Italy

## Abstract

**Background:**

Neurological involvement is a serious complication associated to the surgical removal of impacted mandibular third molars and the radiological investigation is the first mandatory step to assess the risk of a possible post-operative injury to the inferior alveolar nerve (IAN). The aim of this study was to introduce a new radiological classification that could be normally used in clinical practice to assess the relationship between an impacted third molar and mandibular canal on cone beam CT (CBCT) images.

**Material and Methods:**

CBCT images of 80 patients (133 mandibular third molars) were independently studied by three members of the surgical team to draw a classification that could describe all the possible relationships between third molar and IAN on the cross-sectional images. Subsequently, the study population was subdivided according to this classification. The SPSS software, version 15.0 (SPSS® Inc., Chicago, Illinois, USA) was used for the statistical analysis.

**Results:**

Eight different classes were proposed (classes 0-7) and six of them (classes 1-6) were subdivided in two subtypes (subtypes A-B). The distribution of classes showed a prevalence of buccal or apical course of the mandibular canal followed by lingual position and inter-radicular one. No differences have resulted in terms of anatomic relationship between males and females apart from a higher risk of real contact without corticalization of the canal when the IAN had a lingual course for female group. Younger patients showed an increased rate of direct contact with a reduced calibre of the canal and/or without corticalization.

**Conclusions:**

The use of this classification could be a valid support in clinical practice to obtain a common language among operators in order to define the possible relationships between an impacted third molar and the mandibular canal on CBCT images.

** Key words:**CBCT, classification, inferior alveolar nerve, third molars.

## Introduction

Neurological involvement is a serious complication associated to a surgical removal of impacted mandibular third molars. Although the frequency of inferior alveolar nerve (IAN) injures is low, the third molar removal is one of the most common procedure in dental practice so that the absolute number of patients with neurosensory impairment after surgery is significant. For an optimal planning of surgical approach, a radiological investigation is the first mandatory step to assess the risk of a possible post-operative injury to the IAN. Despite the presence of certain radiographic signs on panoramic radiograms (darkening, narrowing or deflection of the root, dark and bifid apex of the root, interruption of cortical outline of mandibular canal, canal diversion or narrowing, island-shaped apex) are mostly associated to a real relationship between the third molar and the mandibular canal, only a cross-sectional CT images obtained by conventional CT or cone-beam CT (CBCT) can define the several types of relationships in a buccal/lingual direction (1). The main drawback of conventional medical CT is the much higher dose that the patient receives in comparison with a panoramic radiography and a higher cost of the examination (2).

Over the last years, CBCT is becoming more common in clinical practice thanks to its spatial resolution and the lower radiation dose as compared to conventional CT. Applications in implantology, endodontics, orthodontics and oral and maxillofacial surgery have been reported (3-6).

Despite the increasing application on CBCT, any radiological classification was introduced to define the possible relationships between third molar roots and IAN course in the buccal/lingual direction. For this reason, the first aim of this technical report was that of introducing a new radiological classification that could be normally used in clinical practice to assess the relationships between an impacted third molar and the mandibular canal on CBCT images. The classification was than applied to study CBCT images of mandibular impacted third molars on a sample of patients that needed extraction. The second aim of this work was that of studying the distribution of impacted third molars in the newly introduced classification.

## Material and Methods

-Patients and evaluation of images

The present observational study was conducted in agreement with the guidelines of the Helsinki Declaration of 1975, as revised in 1983. An informed written consent was obtained from each patient. The study was carried out from April 2013 to September 2013 in the city of Trieste, Italy. On a total of 213 patients consecutively candidated to the surgical removal of one or both mandibular third molars, all of them have shown a close relationship with the mandibular canal on orthopantomography (OPG) were included in this study with no restriction of age or gender. When a real contact between mandibular canal and third molar roots was suspected, in presence of Rood’s signs, the choice of performing CBCT examination was made. Exclusion criteria were pregnancy or impossibility to maintain standing or sitting position.

Finally, 80 patients, 33 males and 47 females with a mean age of 34. 31 years and an age range of 16-80 years, performed the second level radiological examination (CBCT). An oral and maxillofacial surgeon (M.M.), with an oral surgeon (F.C.) and a clinical radiologist with experience in the field of oral and maxillofacial radiology (G.B.), analyzed the CBCT images of the patients for a total of 133 mandibular third molars. The images were acquired by using a CBCT scanner (NewTom VGi, Verona, Italia). The technical parameters used were: 110 kV, 0.3-2 mA, range mAs 2.5- 6.7, scan time <12 s, FOV of 12 x 8 cm or 12 x 15 cm. Voxel size was 0.25 mm and slice thickness of axial images was 0.25 mm. The delivered dose was 2.0-2.2 mGy ± 30%. The images were created in DICOM format and evaluated by axial, cross-sectional and sagittal reconstructions with a thickness of 1 mm and a cutting interval of 1 mm. Images were processed with dental software to create panoramic and sagittal oblique (cross-sectional) reformatted images of the maxilla and mandible.

Subsequently, the images were independently studied by the three members of the surgical team. All the clinicians agreed that the classification had to meet the following requirements (7) thus being:

a. Comprehensive: it must cover all possible relationships between IAN and third molar that may be examined.

b. Easy to use: it has to be simple, logical and reasonable.

c. Acceptable: it should use simple and easy recognizable anatomic landmarks.

d. Reasonable: it has to be mainly designed to estimate the risk of IAN injury and optimize surgical technique

e. Scientifically based: it has to consider the most recent literature knowledge especially the one regarding radiographic signs that are more significantly associated with a IAN injuries (radiographic risk factors).

f. Widely used in clinic: it should be helpful to determine the prognosis and treatment guidelines.

Repeated sessions of discussion have been planned to compare the proposals and to define the final version of the CBCT radiological classification.

-Definition of the radiological classification

When the consensus was reached, the final classification describing the possible IAN/third molar relationships in the buc-cal/lingual direction was defined as follows and summarized in  Table 1,  Table 1 (Cont))

**Table 1:**
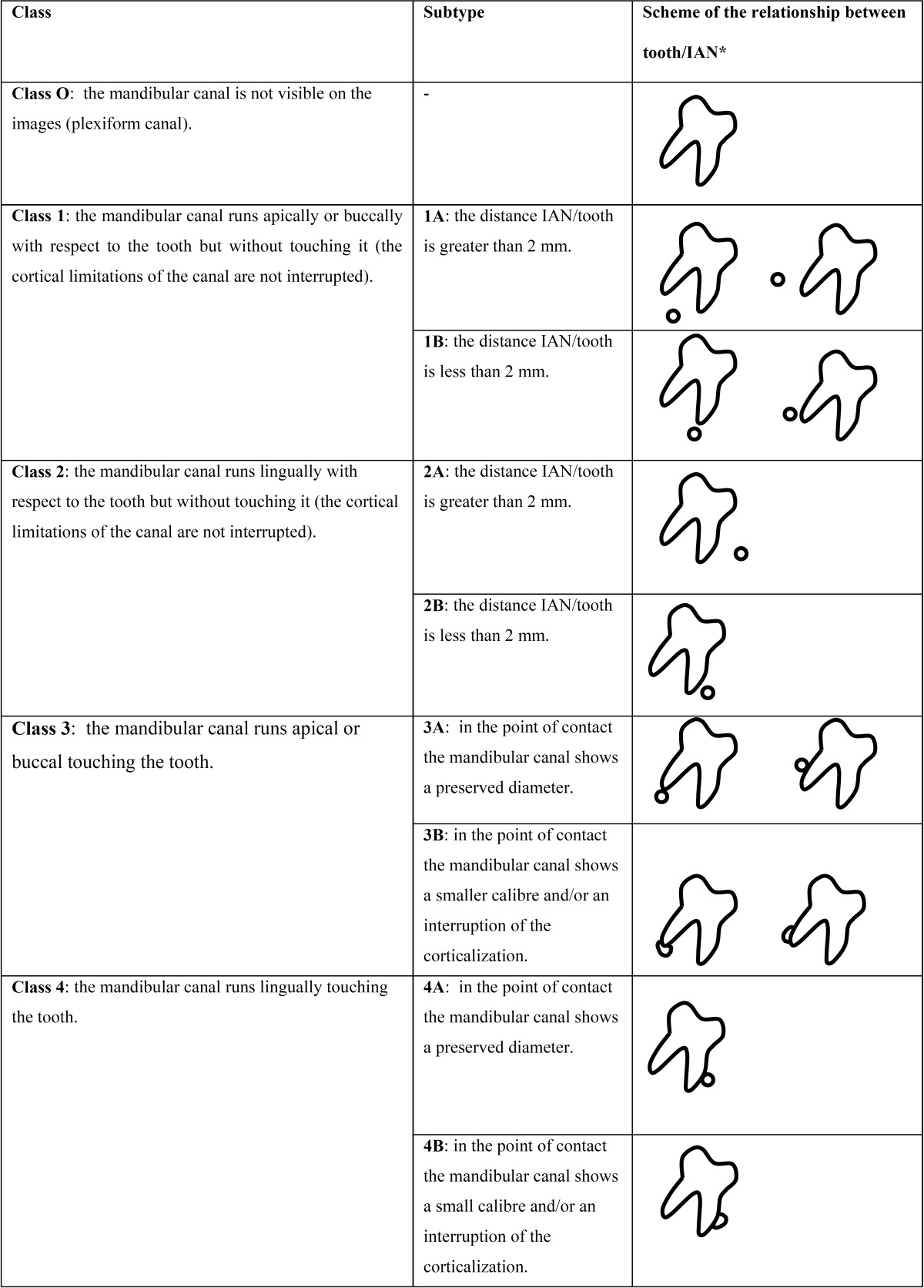
CBCT Radiological Classification for mandibular third molars. Images schematically show the section of a right third molar and its relationship with the mandibular canal in a buccolingual section for each classification types and subtypes.

**Table 1 (Cont):**
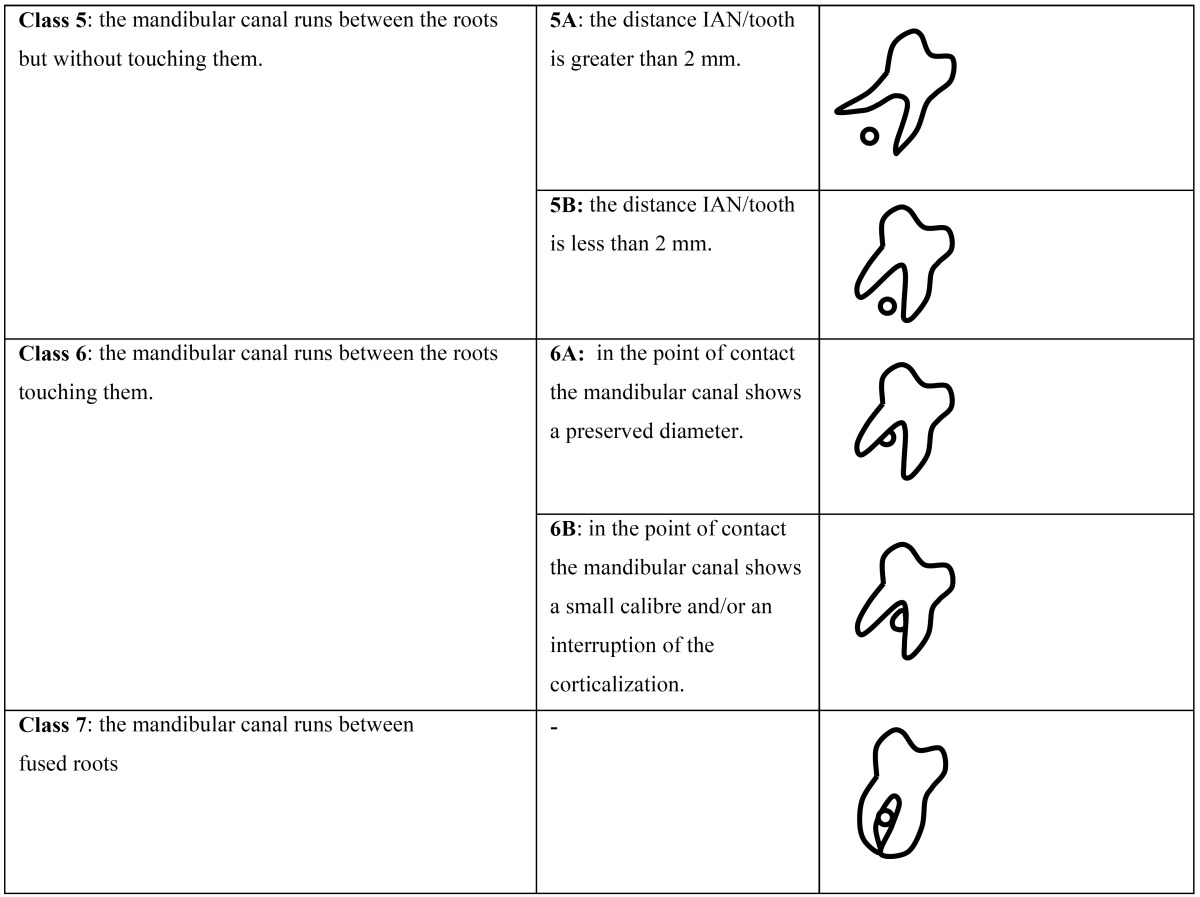
CBCT Radiological Classification for mandibular third molars. Images schematically show the section of a right third molar and its relationship with the mandibular canal in a buccolingual section for each classification types and subtypes.

- Class 0: the mandibular canal is not visible on the images (plexiform canal);

- Class 1: the mandibular canal runs apically or buccally with respect to the tooth but without touching it (the cortical limitations of the canal are not interrupted).

Subtype 1A: the distance IAN/tooth is greater than 2 mm; subtype 1B: the distance IAN/tooth is less than 2 mm;

- Class 2: the mandibular canal runs lingually to the tooth without touching it (the cortical limitations of the canal are not interrupted).

Subtype 2A: the distance IAN/tooth is longer than 2 mm; subtype 2B: the distance IAN/tooth is less than 2 mm;

- Class 3: the mandibular canal runs apical or buccal touching the tooth.

Subtype 3A: in the point of contact the mandibular canal shows a preserved diameter; subtype 3B: in the point of contact the mandibular canal shows a smaller calibre and/or an interruption of the corticalization;

- Class 4: the mandibular canal runs lingually touching the tooth.

Subtype 4A: in the point of contact the mandibular canal shows a preserved diameter; subtype 4B: in the point of contact the mandibular canal shows a smaller calibre and/or an interruption of the corticalization;

- Class 5: the mandibular canal runs between the roots but without touching them.

Subtype 5A: the distance IAN/tooth is greater than 2 mm; subtype 5B: the distance IAN/tooth is less than 2 mm;

- Class 6: the mandibular canal runs between the roots touching them.

Subtype 6A: in the point of contact the mandibular canal shows a preserved diameter; subtype 6B: in the point of contact the mandibular canal shows a smaller calibre and/or an interruption of the corticalization;

- Class 7: the mandibular canal runs between fused roots

Finally, the study population was subdivided according to the classification.

-Statistical analysis

The SPSS software, version 15.0 (SPSS® Inc., Chicago, Illinois, USA) was used for the statistical analysis.

The Cohen K values were calculated for inter-observer agreement.

To assess the difference in the frequency of the classification classes and subtypes between male and female groups the exact Fisher’s test was used. The difference in age distribution among classes was tested by univariate ANOVA and post-hoc Bonferroni test was used for the pairwise comparisons. The exact Fisher’s test was used to find differences in the distribution of cases with contact between IAN and roots when the course of the IAN was buccal/apical, lingual or interradicular.

## Results

In the assessment of classes and subtypes on CBCT images, inter-observer agreement ranged from good to excellent (K value range: 0.67-0.88).

 Table 2shows the distribution of classes and subtypes in the whole study population. The most represented classes were 3B (24%), 4B (21%) and 3A (19.5%). When data were split by gender, the most populated class was 3B for males (42.4%) and 4B for females (46.8%). No differences were observed in the distribution of classes in male and female groups, except for class 4B (Fisher exact test; *p*<0.005). No cases were found for classes 0 and 7. The highest mean age was observed for class 1A (42.9 ± 2.7 years) while the lowest one was found in class 6A (18 years). Statistical analysis showed a significant difference for classes 6B (20.5 ± 0.9 years) and 1B (23.9 ± 0.9 years) in respect with class 1A (post-hoc Bonferroni test, *p*<0.001).  Table 3 shows that on a total of 133 third molars, 92 had a direct contact with IAN while 41 had not. The difference was statistically significant (Fisher exact test; *p*< 0.05). The presence or absence of a direct contact with roots was also matched with prevalence of buccal/apical, lingual and interradicular course of the mandibular canal. The most frequent anatomical course of the IAN, when not in contact with the third molar, was buccal or apical. Only one case showed a lingual course and no cases were found for the interradicular one. When a direct contact between IAN and the tooth was observed, the course was mainly buccal or apical but a significant higher amount of cases showed a lingual course, compared to the “no contact” group (Fisher exact test; *p*< 0.001).

**Table 2:**
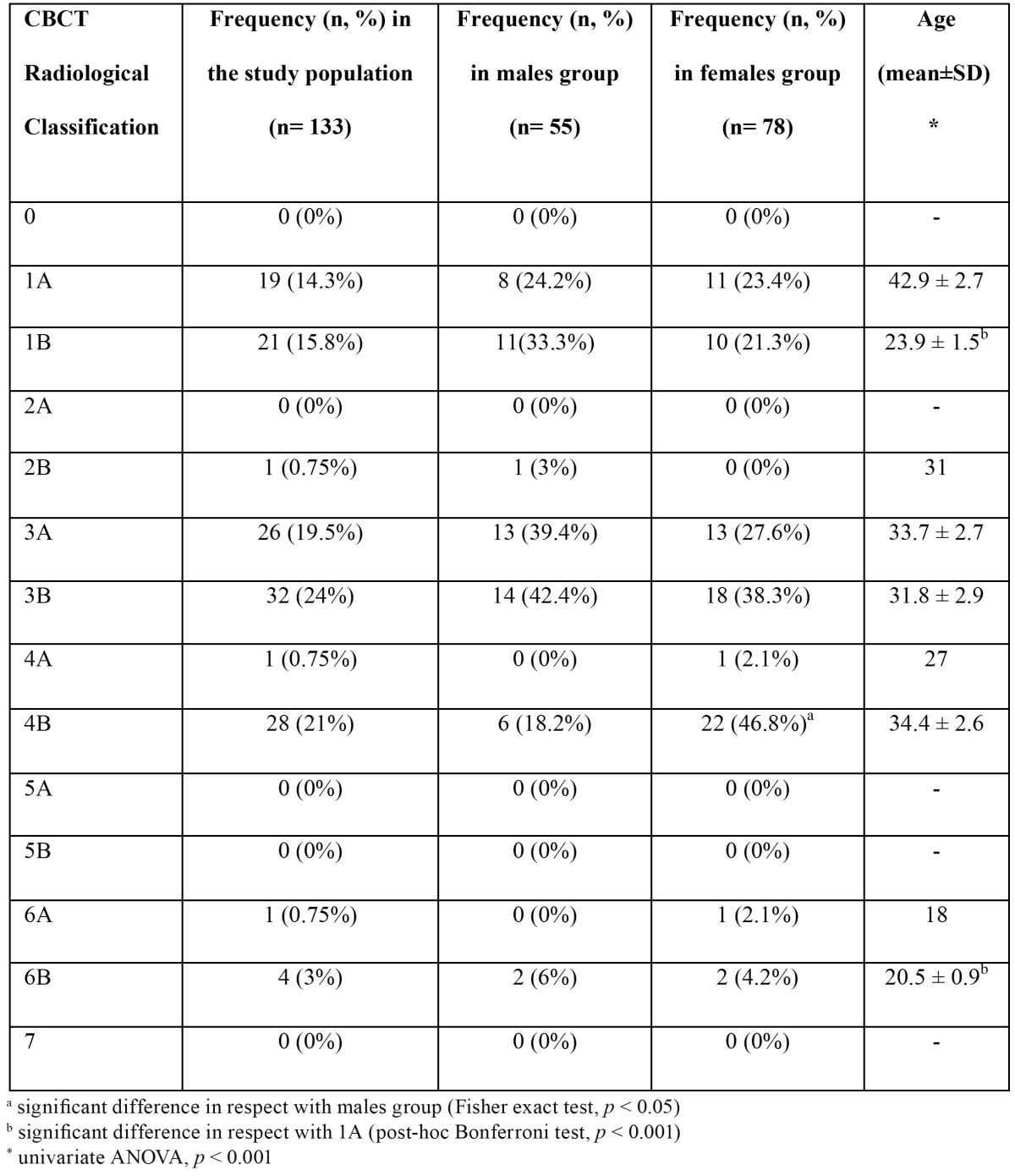
Frequency of Classes and Subtypes of the CBCT Radiological Classification in the study sample.

**Table 3:**
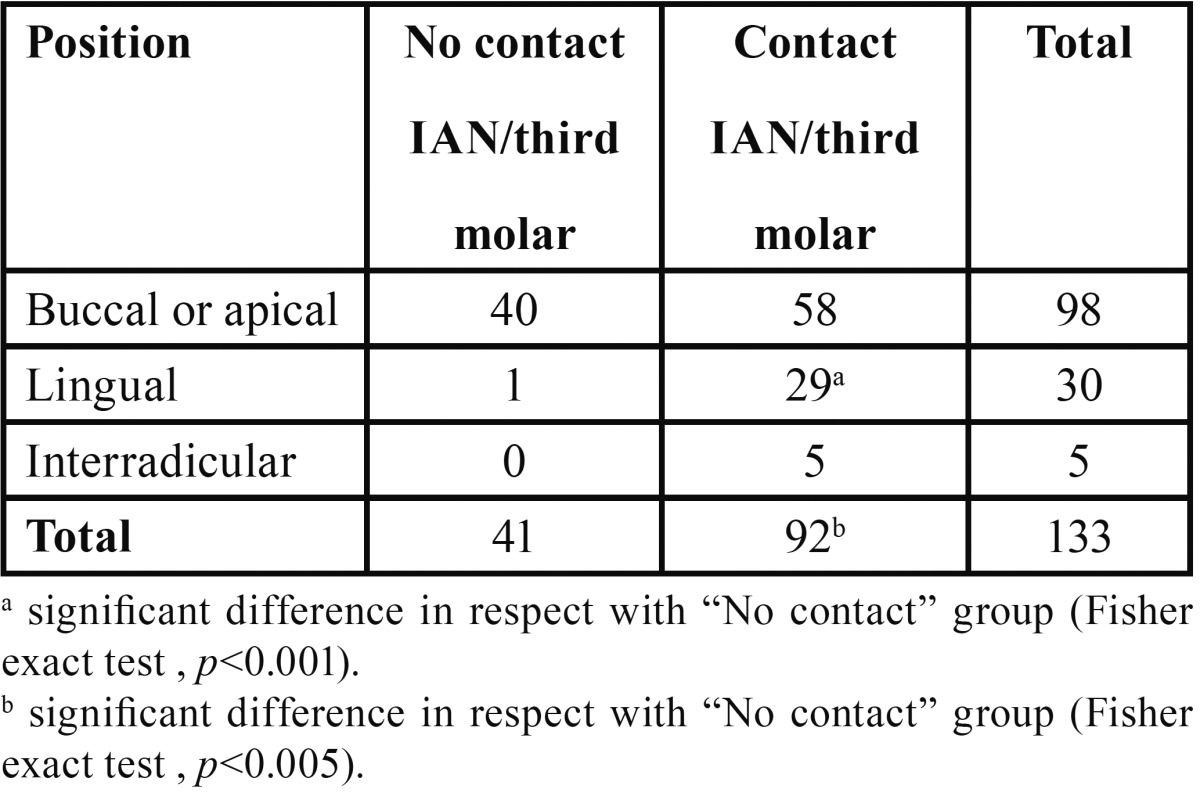
Course of the mandibular canal in respect with the impacted third molar. The presence or absence of a direct relationship is shown.

## Discussion

Several studies have been conducted on risk factors and complications associated with the surgery of impacted third molars (8-10). Although the relatively low percentage of post-operative complications occurred, the lesion of the IAN is the one that is mostly associated to patient discomfort and legal contentious. To intercept and predict the risk of nerve injury, the radiographic examination is routinely performed in clinical practice before extraction.

Actually, two radiographic classifications, the first one introduced in 1926 by Winter, the second one by Pell and Gregory in 1933, are still the most used to define the grade of inclusion of upper and lower third molars on OPG. Winter classified the third molar considering its inclination with respect to the major axis of a normally inclined second molar so that the wisdom tooth can be: mesio-inclined, vertical or normally inclined, disto-inclined, horizontal, inverted. The Pell and Gregory classification considers classes I, II, and III and A, B, and C based on the position of the inferior third molar with respect to the mandibular bone and second molar occlusal plane. Upper molars are classified as belonging to class A, B, or C with respect to second molar occlusal plane (11).

However, while these classifications can predict the difficulty of the surgery, they do not provide any information regarding the relationship of the tooth with the mandibular canal and the risk of neurological involvement. If no signs of close relationship are observed, the anatomic information obtained with OPG is sufficient to plane the surgical technique (1). Otherwise, when OPG shows an anatomic intimacy between the third molar and the mandibular canal or when specific radiographic signs (darkening, narrowing or deflection of the root, dark and bifid apex of the root, interruption of the cortical outline of the mandibular canal, diversion or narrowing of the canal, island-shaped apex) are detected on the radiogram, when possible a CT or CBCT examination has to be performed to confirm the real presence and eventually the type of the relationship on a buccal/lingual section (1,12-14). CT and CBCT has the capability of providing images in any direction and orientation, however the coronal sections are considered to be the most useful ones because these projections add further information that would not be appreciable on OPG, for example the number of roots and the root morphology (15-17).

To identify the different types of possible relationships between the third molar and the mandibular canal, a new radiological classification applicable on the cross-sectional images was proposed. The rationale of this classification is that of researching a restricted number of categories able to describe all the possible anatomic variants that the clinician can encounter before surgery. If possible, the classification has to suggest to the oral surgeon the optimal surgical technique and provide a progressive rate of risk of IAN injury. Finally, the anatomic landmarks have to be simply recognizable on the CT or CBCT images so that the classification types and subtypes can be easily identified.

Three main argumentations of the recent literature were considered during the conceptualization of the classification.

The first one regards the importance of the IAN as regards the tooth and to the vestibular/lingual plates on a buccal/lingual direction (vestibular, lingual, apical or inter-radicular localization). CT or CBCT cross-sectional reconstructions provide this funda-mental information for a precise planning of the extraction. The knowledge of the canal course can indeed suggest the optimal directions and the entity of forces to apply during luxation, tooth sectioning or ostectomy. The positional relation between the third molar and mandibular canal has been recognized as a possible predictive factor of IAN injure, with a major risk for the lingual-sided canals (18). On a sample of 53 third molars extracted, Ghaeminia *et al.* (19) found that the IAN was more frequently exposed when the mandibular canal was situated at the lingual side or interradicular to the third molar roots than buccally (*p* < 0.02). Furthermore, they observed that in all patients with sensory impairments, the mandibular canal was positioned lingual to the third molar roots as seen on CBCT images (*p* < 0.02). This could be because the surgeon starts his surgical approach on the vestibular side, generating unfavourable lingually directed forces (19). This background justified the choice of subdividing classes basing on IAN course (buccal/apical or lingual).

The second point was the importance of the distance between IAN and third molar. Up to now, few studies have quantified the minimal distance between mandibular canal and the impacted third molar which significantly increases the risk of neurological damage. Jhamb *et al.* (15) divided the measured distance in 4 categories, > 1 mm, 0 to 1 mm, 0 mm and 0 mm with cortical break. They found cases of IAN paresthesia only for the 0 mm category with cortical break. However, a certain rate of risk can be observed also when the distance is higher. Infact, physical, toxic, ischemic, inflammatory processes, act as principal factor or co-factor in developing of peripheral neuropathy (20-21). In particular, the laceration of vasa vasorum or compression of nerve fibres due to the force applied during extraction or to the postsurgical edema, can elicit a neuropraxia. Sammartino *et al.* (20) proposed a safety distance from IAN of 1.5 mm during implant placement to avoid indirect lesions of the nerve bundle. This is the reason why a cut-off of 2 mm was chosen in our classification as an acceptable distance to differentiate cases with higher risk of indirect lesion (distance > 2 mm) from those with a lesser one (distance < 2 mm).

The third aspect that was considered is the presence/absence of the corticalization of the mandibular canal. The loss of cortical integrity and the size of cortical defect were associated to an increased risk of IAN injury (22). Susarla *et al.* (23) estimated that cortical interruption was associated to increased odds of IAN exposure (odds ratio of 12.8). When a real relationship with the IAN occurs, paresthesia can reach an incidence of 35.6% (15). Considering this aspect, a further differentiation was made in the classification between third molars presenting a direct contact with IAN but without narrowing or decorticalization of the canal and teeth in contact with a preserved mandibular canal (preserved calibre and corticalization).

Consequently, eight different classes have been proposed and six of them have been subdivided in two categories. The K value showed a good or excellent inter-observer concordance with a minimum of 0.67 for the class 1B and a maximum of 0.88 for the class 1A.

Distribution of the classes showed a higher frequency of 3B grade (24% of patients) followed by 4B (21%) and 3A (19.5%) grades.

 No cases were found for 0, 2A, 5A, 5B, 7 grades. This finding was expected for classes 0 and 7 considering that plexiform canal or fused root surrounding the IAN are very rare situations. A possible explanation of the absence of cases for class 2A has to be researched in the reduced thickness of the alveolar lingual bone in correspondence of third molar. A smaller space forces the canal to approach molar roots (class 2B) or, more often, to touch the tooth (classes 4A and 4B). For the same assumption, no cases were observed for 5A and 5B classes: a reduced inter-radicular space promotes a relation of contiguity between the IAN and the roots (classes 6A and 6B). However, the frequency of 6A and 6B classes is very low, probably for the relatively high percentage of fused or single roots often associated to impacted mandibular third molars (24).

Age was not equally distributed among classes (Table 2). Class 1A was associated to the higher mean age while classes 1B, 6A and 6B showed the lesser ones (univariate ANOVA, *p* < 0.001). The difference was statistically significant for 1B and 6B classes in respect with class 1A (post-hoc Bonferroni test, *p* < 0.001). The incidence of a real contact between third molar and IAN was much more frequent in the third decade of life. This finding agrees with observation of De Melo et al. (1) and suggests that a greater risk of injury is expected for extraction in young adult especially before age of 30. Considering gender, no differences were observed in the distribution of classes with the exclusion of 4B grade. This result demonstrates that in women, the lingual course of IAN is more associated to an intimate relationship with molar roots than in men. One of the reasons of this closer relationship has to be found in the buccal/lingual thickness of the mandibular bone. As observed by Nakagawa *et al.* (25), with a thinner mandible in women, less distance is likely to be seen between tooth and IAN.

The distribution of the classes showed a prevalence of buccal or apical course of the mandibular canal followed by lingual position and inter-radicular one. These results are in accordance with the greatest part of the consulted literature (1,18). When data were matched with the presence or absence of contact between IAN and third molar, irrespective of corticalization of the canal or not, a significant difference was observed for the lingual course ( Table 3). Also the inter-radicular position showed the same tendency but this result was not amenable to tests of inference. Our results agrees with those obtained by Jhamb *et al.* (15) that found that the absence of cortication of the canal was more frequent when the canal had a lingual or inter-radicular course.

The objective of this study was to propose a new classification for impacted mandibular third molars on CBCT images. To our knowledge, this is the first systematic classification that identifies all the possible relationships between third molar and IAN.

However, the aim of the classification was not merely to detect if a real relationship between the mandibular canal and the roots of the third molar exists, but to intercept the individual anatomical relations for an optimised surgery. Classification has been applied to study an initial sample of 133 impacted third molars. Results highlighted that no differences exist in terms of anatomic relationships between males and females apart from a major risk of real contact without corticalization of the canal when the IAN has a lingual course for female group. Younger patients showed an increased rate of direct contact with a reduced calibre of the canal and/or without corticalization. If taken as preliminary findings of an uncontrolled, exploratory study, we might conclude that the patients at high risk of developing a IAN damage are young woman belonging to the third decade with a lingual course of the mandibular canal.

The study has some limitations that should be further considered. First, some classes had no cases because of the rarity of the anatomic relationship suggesting that a greater study population will be necessary to confirm the trends observed. Moreover, the study does not provide any data regarding the correlation between class and clinical results in terms of IAN injury and periopera-tive complications. In this sense, further studies will be necessary.

The application of the classification is strongly encouraged in clinical practice to obtain a common language among clinicians (oral and maxillofacial surgeons, clinical radiologists) in defining an impacted third molar on CBCT images.
